# Laparoscopic minimal incision at cystic duct confluence for common bile duct stones in patients with thin-caliber bile ducts: A retrospective comparative study

**DOI:** 10.1097/MD.0000000000048887

**Published:** 2026-05-15

**Authors:** Yifeng Huang, Jiansen Xie, Gengsheng He

**Affiliations:** a Department of Hepatobiliary Surgery, Longhua District People’s Hospital, Shenzhen, Guangdong, China.

**Keywords:** laparoscopy, mini-incision at the confluence, primary suture, thin common bile duct

## Abstract

To investigate the clinical utility of minimally invasive laparoscopic choledocholithotomy via the cystic duct confluence approach for secondary stones in slender common bile ducts. Data from 182 patients (74 male, 108 female; median age 45 years, range 32–68) with gallbladder calculus and secondary stones in slender common bile ducts (6–8 mm diameter), treated at Longhua District People’s Hospital, Guangdong, China from September 2021 to June 2024, were retrospectively analyzed. Of these, 90 underwent laparoscopic choledocholithotomy via cystic duct confluence with primary sutures (minimally invasive group), while 92 underwent laparoscopic common bile duct exploration with T-tube drainage (T-tube group). Clinical data and perioperative indicators were compared between groups. In carefully selected patients with secondary stones in slender common bile ducts, the laparoscopic minimally invasive choledocholithotomy via the cystic duct confluence approach is a favorable option, enabling rapid recovery and meriting broader application. Clinical data for the 2 groups were comparable (*P* > .05). The minimally invasive group had a mean operative duration of 95.67 ± 25.56 minutes, intraoperative hemorrhage of 10.0 mL (5.0–10.0 mL), and Winslow’s foramen drainage tube indwelling time of 4.47 ± 1.25 days. The stone clearance rate in the minimally invasive group was 100% (90 out of 90 patients). The T-tube group had an operative duration of 103.87 ± 29.83 minutes, intraoperative hemorrhage of 10.0 mL (5.0–10.0 mL), and Winslow’s foramen drainage tube indwelling time of 3.76 ± 1.06 days. The stone clearance rate in the T-tube group was 100% (92 out of 92 patients). There were no statistical differences between the groups (*P* > .05). Compared with the T-tube group, the minimally invasive group experienced significantly shorter postoperative hospital stay (7.72 ± 1.63 vs 12 ± 2.45 days), lower treatment costs (1.75 ± 0.27 × 10^4^ vs 2.16 ± 0.36 × 10^4^ CNY), exhibited reduced postoperative numerical rating scale pain scores (1.56 ± 0.67 vs 4.13 ± 0.79), and earlier postoperative time to ambulation (8.34 ± 1.67 vs 15.56 ± 3.83 hours; *P* < .05). Both groups had comparable bile leakage rates (1.11% vs 1.08%; *P* > .05), with no cases of residual stones or biliary strictures.

## 1. Introduction

Gallbladder calculi with secondary choledocholithiasis is a common disease treated with hepatobiliary surgery. Current mainstream treatment methods include endoscopic sphincterotomy combined with laparoscopic cholecystectomy (LC), and laparoscopic common bile duct exploration (LCBDE) combined with LC. Traditional T-tube drainage after bile duct exploration is associated with complications, such as postoperative bile loss, pain at the tube site, and risk for accidental tube dislodgement, which affect patient quality of life and has led to the increasingly widespread clinical application of primary bile duct closure after common bile duct exploration. Although there is currently no unified standard regarding the required bile duct diameter for primary closure of the common bile duct. Currently, most scholars define the small-caliber common bile duct as having a diameter of 6 to 8 mm,^[1,2]^ and when performing anterior choledochotomy exploration and primary closure, 1 should be vigilant against serious complications such as bile duct stricture. Therefore, in recent years, minimally invasive stone extraction via the confluence of the cystic duct with primary closure has attracted increasing attention from biliary surgeons. In recent years, the minimally invasive lithotomy through the confluence of the cystic duct with primary suture has attracted attention. From an anatomical perspective, this method avoids the anatomical operations on the hepatoduodenal ligament and the incision of the common bile duct, preserves the integrity of the biliary system, and reduces the risk of damaging adjacent structures. From a physiological perspective, this method does not require the placement of a T-tube for drainage, thus avoiding bile loss and maintaining normal bile circulation and digestive function. Meanwhile, it also avoids the pain at the tube – placement site, reduces the body’s stress response, helps the patient’s physiological functions recover more quickly, and reduces the systemic inflammatory response.

The present study aimed to explore the safety and efficacy of minimally invasive stone extraction via the cystic duct confluence with primary closure to promote rapid postoperative recovery. We retrospectively analyzed clinical data from patients who underwent laparoscopic minimally invasive stone extraction via the cystic duct confluence with primary closure at the Longhua District People’s Hospital (Guangdong, China) between September 2021 and June 2024.

## 2. Materials and methods

### 2.1. General information

Clinical data from 182 patients (74 male, 108 female; 32–68 years of age; median age, 45 years) with calculus of the gallbladder combined with fine-diameter choledocholithiasis, admitted to Longhua District People’s Hospital (Guangdong, China) between September 2021 and June 2024, were retrospectively analyzed. forty-four patients had concurrent diabetes mellitus. All patients underwent routine biochemical and magnetic resonance cholangiopancreatography (MRCP) examinations before surgery, with thorough evaluations of the diameter of the cystic duct, number of choledocholiths, long diameter of the stones, diameter of the common bile duct, presence or absence of stones in the cystic duct, and any biliary tract variations. such as *Parazacco spilurus* subsp. spilurus.

### 2.2. Inclusion and exclusion criteria

The inclusion criteria were as follows: MRCP clearly revealing calculus of the gallbladder combined with choledocholithiasis; preoperative MRCP imaging data revealing a common bile duct diameter of 6 to 8 mm; and no significant organ dysfunction or coagulation disorders.

The exclusion criteria were as follows: presence of obstructive jaundice; combined calculus of the intrahepatic bile duct; variant cystic duct confluence *P spilurus* subsp. spilurus: ultra-low confluence, left-sided confluence, posterior confluence, etc; preoperative nutritional assessment numerical pain rating scale (NRS) 2002 score ≥3; and contraindications to laparoscopic surgery.

### 2.3. Protocol

Grouping: patients were divided into groups according to surgical method. Those who underwent laparoscopic trans-cystic duct confluence mini-incision for biliary stone extraction with primary sutures were designated as the mini-incision group (n = 90), whereas those who underwent LCBDE with T-tube drainage were designated as the T-tube group (n = 92). Patients in both groups underwent outpatient imaging examinations at 1 month after surgery to evaluate stone clearance; patients in the microincision group were assessed by MRCP, while those in the T-tube group underwent T-tube cholangiography. Outpatient follow-up visits were conducted again at 6 and 12 months after surgery. Both groups completed liver function tests and MRCP examinations to evaluate residual stones. If patients could not return to the hospital for reexamination due to special circumstances, they could undergo reexamination at local hospitals, and the reexamination results were obtained by contacting the patients.

Instruments: a high-definition laparoscopic system (Karl Storz, Tuttlingen, Germany), Micro-Tech disposable choledochoscope (outer diameter, 3 mm), a disposable stone retrieval basket, and microelectrode lithotripsy device were used for surgery.

Surgical personnel: all surgeries were performed by the same surgical team.

**Figure 1.**
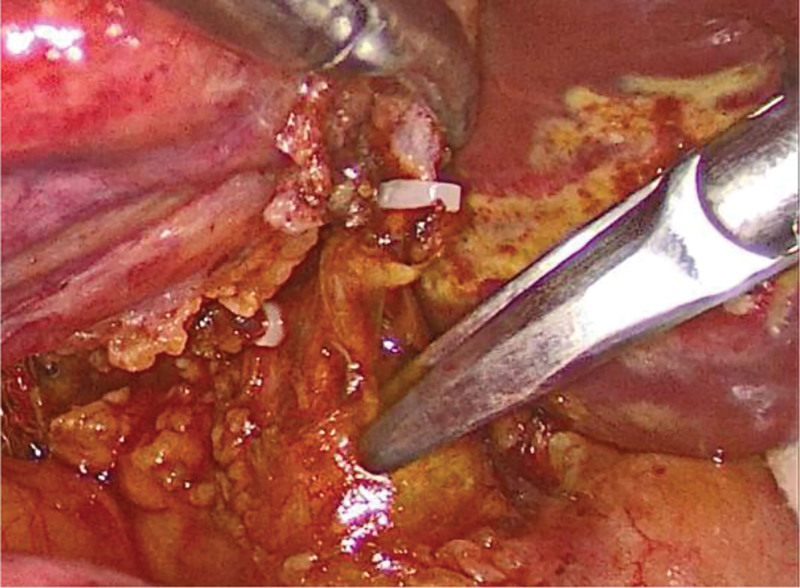
The cystic duct is incised longitudinally with tissue scissors.

**Figure 2.**
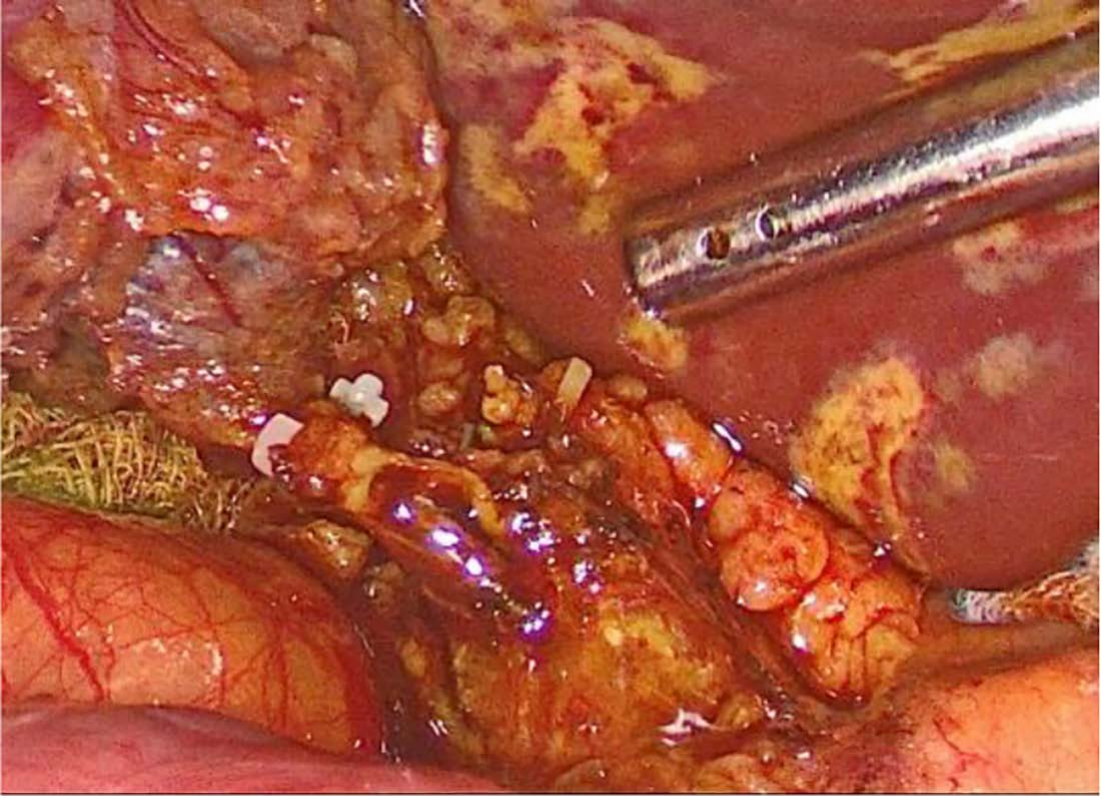
The confluence of the cystic duct is sharply incised for 2 to 3 mm.

**Figure 3.**
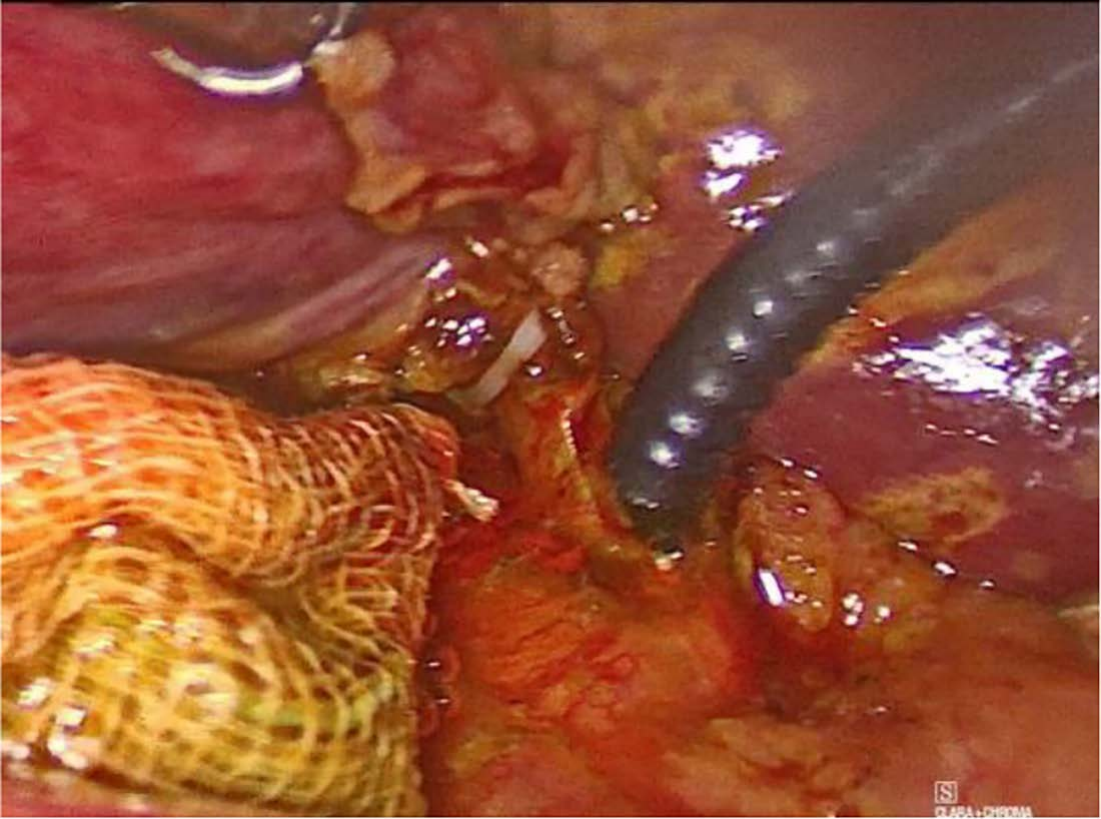
A disposable ultra-slim choledochoscope is inserted through the incised confluence for biliary tract exploration.

**Figure 4.**
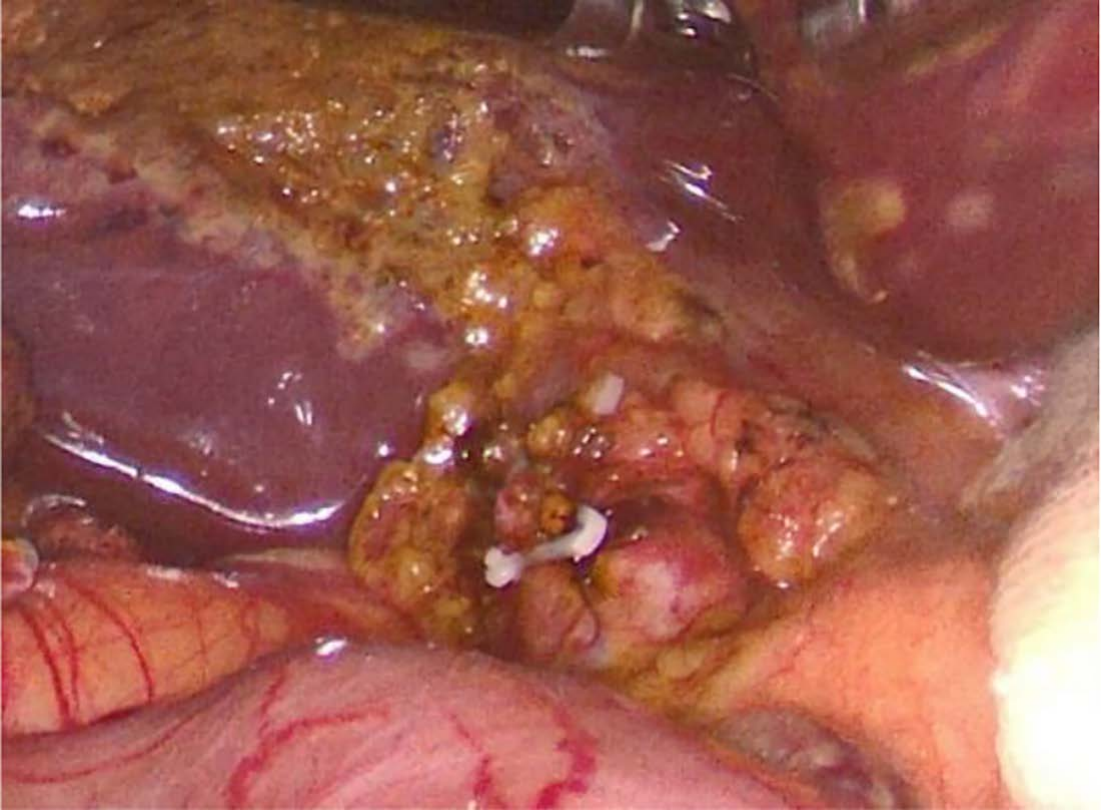
The incision is closed with absorbable sutures, and the cystic duct stump is clipped with a ligating clip.

Microincision group: conventional 4-port method. After establishing pneumoperitoneum, atraumatic grasping forceps and an electrocautery hook were inserted through the auxiliary and main operating ports, respectively. Using a combined antegrade-retrograde approach, an electrocautery hook was used to coagulate and incise the serosa along the right edge of the hepatoduodenal ligament. Calot’s triangle was dissected into thin layers to isolate the cystic artery and duct. The hepatoduodenal ligament was dissected to expose the common hepatic and bile ducts, clearly identifying the confluence of the 3 ducts (*Broussonetia papyrifera*). Subsequently, the cystic arteries were clipped and divided. The gallbladder fundus was grasped with atraumatic forceps and an electrocautery hook was used to dissect along the plane between the gallbladder seromuscular layer and mucosa (approximately 0.5 cm) from the liver edge, completely separating the gallbladder from the gallbladder bed. Hemostasis of the gallbladder bed was achieved using bipolar electrocautery. The cystic duct was skeletonized at the confluence, and a single clip was applied to the gallbladder side of the cystic duct. The lumen of the cystic duct was transversely incised using scissors, followed by a longitudinal incision along the long axis to the confluence with the common bile duct (Fig. 1). The right side of the common bile duct was opened approximately 2 to 3 mm along the direction of the cystic duct confluence (Fig. 2). A single-use choledochoscope was used to explore the biliary tract, and bile duct calculi were removed using a stone retrieval basket or in combination with lithotripsy devices (Fig. 3). Sphincter of Oddi function was intact, with no residual stones. The intrahepatic bile ducts were explored, and no stones or neoplasms were detected within the accessible range, with no inflammatory edema in the bile duct wall. The common bile duct incision was continuously sutured to full thickness using 5-0 PDS-II from the lateral wall toward the cystic duct. The cystic duct stump was clipped approximately 5 mm from the confluence and completely divided (Fig. 4). A custom-made specimen bag with a latex glove was used to place the resected gallbladders. The operative field was irrigated, and scattered fluids in the abdominal cavity were aspirated. The gauze pressed against the operative field showed no bile staining. The specimen bag and the gallbladder were removed through the subxiphoid port. A drainage tube was placed in the foramen of Winslow and exteriorized through the right port.

T-tube group: the gallbladder was removed using the same antegrade-retrograde combination method. A 5 to 8 mm longitudinal incision was made in the supraduodenal segment of the bile duct. A choledochoscope was inserted through a subxiphoid incision. After confirming complete stone removal using the same method, a 16 Fr T-tube was placed. The bile duct incision was intermittently sutured using absorbable sutures on both sides of a T-tube. The tube was exteriorized through a puncture hole along the midclavicular line. No leakage was observed during the water-injection test. A drainage tube was placed in the foramen of Winslow and exteriorized through the right puncture hole. The remaining procedures were the same as those described above.

### 2.4. Observation indicators

Operative duration, hemorrhage volume, length of hospital stay, treatment costs, postoperative 11-point NRS score (scored 0–10), time to ambulation, duration of abdominal drainage tube placement, incidence of bile leakage, residual stones, and biliary stricture were compared between the 2 groups.

### 2.5. Statistical processing

Statistical analyses were performed using SPSS version 22.0 (IBM Corporation, Armonk). Patient age, common bile duct diameter, cystic duct diameter, and perioperative clinical indicators conformed to a normal distribution, with measurement data expressed as mean ± SD and compared using the *t*-test. Intraoperative hemorrhage volume exhibited a skewed distribution, with data expressed as median (interquartile range [IQR], i.e., Q_1_, Q_3_), and comparisons between the groups were performed using the rank-sum test. Postoperative ambulation time, postoperative pain, bile leakage, and other complications were categorical data expressed as n (%) and compared using the χ^2^ test. Differences with *P* < .05 were considered to be statistically significant.

### 2.6. Ethical approval

This research protocol has been reviewed and approved by the Ethics Committee of Shenzhen Longhua District People’s Hospital. Ethical Approval Number: LHRY-LLS(Yan)[2025]No.(104).

## 3. Results

### 3.1. Comparison of general data

Two groups were compared in terms of age, cystic duct diameter, presence of cystic duct stones or diabetes mellitus, number of common bile duct stones, and common bile duct diameter, with *P* > .05 (Table 1).

**Table 1:** Comparison of general data between the 2 groups of patients.

Project	Mini-incision group (n = 90)	T-tube group (n = 92)	Statistic	*P*-value
Age (yr)	48.6 ± 14.5	49.8 ± 15.2	*t* = 2.462	.104
Gender (n, %)			*Χ*^2^= 3.787	.097
Male	36 (40.0%)	38 (41.3%)		
Female	54 (60.0%)	54 (58.7%)		
Cystic duct diameter (mm)	4.2 ± 1.4	4.0 ± 1.2	*t* = 1.786	.623
Calculus of cystic duct (n, %)	9 (10.0%)	12 (13.0%)	*Χ*^2^ = 1.395	.768
Diabetes mellitus (n, %)	22 (24.4%)	22 (23.9%)	*Χ*^2^ = 2.784	.834
Choledocholithiasis quantity			*Χ*^2^ = 3.566	.723
Solitary stones	34 (37.8%)	36 (39.1%)		
Multiple stones	56 (62.2%)	56 (60.9%)		
Common bile duct diameter (mm)	7.3 ± 0.5	7.1 ± 0.6	*t* = −0.345	.867

### 3.2. Comparison of perioperative data

Compared with the T-tube group, the microincision group showed no statistically significant difference in operative time, intraoperative blood loss, and abdominal drainage tube indwelling time (*P* > .05); the microincision group had earlier postoperative ambulation time, shorter length of hospital stay, and lower total treatment cost than the T-tube group, with statistically significant differences (*P* < .05; Table 2).

**Table 2:** Comparison of perioperative conditions between the 2 groups of patients.

Group	Operation time (min)	Blood loss during surgery (mL)	Abdominal cavity drainage tube indwelling time (d)	Time of getting out of bed after surgery (h)	Length of hospital stay (d)	Total treatment cost (CNY)
Mini-incision group	95.67 ± 25.56	10.0 (5.0,10.0)	4.47 ± 1.25	8.34 ± 1.67	7.72 ± 1.63	1.75 ± 0.27 × 10^4^
T-tube group	103.87 ± 29.83	10.0 (5.0,10.0)	3.76 ± 1.06	15.56 ± 3.83	12 ± 2.45	2.16 ± 0.36 × 10^4^
Statistic	*t* = 1.574	*Χ*^2^ = −0.217	*t* = 1.865	*t* = 2.756	*t* = 2.173	*t* = 2.597
*P*-value	.163	.967	.756	.026	.041	.038

### 3.3. Postoperative complications

One case of biliary fistula occurred postoperatively in both groups of patients (*P* > .05). Comparison of postoperative NRS-11 pain scores between the 2 groups showed that the microincision group had a score of (1.56 ± 0.67), while the T-tube group had (4.13 ± 0.79; *P* < .05). All patients were followed up regularly after discharge for 6 to 12 months, and no residual stones or biliary stricture complications occurred in either group (Table 3).

**Table 3:** Comparison of common postoperative complications between the 2 groups of patients.

Group	Biliary fistula (n, %)	postoperative 11-point NRS score (NRS-11)	Stone residue (n, %)	Biliary stricture (n, %)
Mini-incision group	1 (1.11%)	1.56 ± 0.67	0	0
T-tube group	1 (1.08%)	4.13 ± 0.79	0	0
Statistic	*Χ*^2^ = 1.134	*t* = −4.543	NA	NA
*P*-value	.347	<.001	NA	NA

NRS = numerical pain rating scale.

## 4. Discussion

Cholelithiasis is one of the most common diseases treated with hepatobiliary surgery. In recent years, many patients with calculus of the gallbladder have adopted a relatively conservative attitude toward treatment options, leading to 10% to 15% of patients with gallbladder calculus presenting with secondary choledocholithiasis at the time of consultation.^[3]^ Among these, some patients exhibit short calculi of the gallbladder migrating into the bile duct, with relatively small stones, and exhibit no signs of cholangitis on the initial visit. With the widespread adoption of MRCP diagnostic techniques, the detection rate for choledocholithiasis without cholangiectasis (i.e., small-diameter choledocholithiasis) has increased annually. The diameter of these bile ducts typically ranges from 6 to 8 mm.^[4-6]^

Various surgical approaches are available for treating such patients, with ERCP + LC and LCBDE + LC the most common.^[7]^ Due to the potential for permanent damage to duodenal papillary function during endoscopic retrograde cholangiopancreatography procedures, particularly in younger patients, many surgeons prefer the LCBDE + LC approach.^[8]^ Traditional laparoscopic primary closure via anterior choledochotomy carries a high risk for postoperative stricture.^[9,10]^ In recent years, reports have highlighted the significant clinical efficacy of common bile duct exploration via the cystic duct route, although this method is limited by cystic duct diameter, insertion angle, as well as the size and number of choledocholiths.^[11,12]^

Relevant clinical studies have leveraged the anatomical advantage of the dilated cystic duct confluence to significantly improve the success rate of small-diameter common bile duct exploration.^[13]^ With the development and clinical application of ultrafine disposable choledochoscopes, combined with insights from domestic research and the literature, our team has adopted a minimally invasive approach involving a microincision at the cystic duct confluence combined with ultrafine choledochoscopy for treating small-diameter choledocholithiasis in recent years, achieving favorable clinical outcomes.

After comparison of the clinical data of the patients included in the study between the 2 groups, *P* > .05, indicating that the 2 groups were comparable. There were no significant differences in operative duration, intraoperative hemorrhage volume, or postoperative complications between the traditional T-tube and microincision groups, indicating that exploration via microincision at the cystic duct confluence did not increase surgical difficulty or risk. Analysis of postoperative NRS-11 pain scores between the 2 groups (*P* < .05) revealed that postoperative pain in the T-tube group was significantly more severe than that in the microincision group. Due to the impact of pain and psychological factors associated with accidental tube dislodgement, most patients in the T-tube group were unwilling to get out of bed early, which hindered their postoperative rapid recovery and resulted in a prolonged hospital stay. Furthermore, after being discharged with the tube, patients in the T-tube group required return to the hospital 8 weeks postoperatively for T-tube cholangiography or choledochoscopy to assess the suitability for T-tube removal. This not only affected patients’ daily life and work but also led to a significant increase in treatment costs.^[14,15]^ In terms of complications, 1 case of bile leakage occurred in each group, both of which were cured after unobstructed drainage, with no severe complications observed. Follow-up revealed no residual stones or long-term biliary strictures, demonstrating that microincision is safe and feasible when suitable surgical candidates are selected. We found that this approach offers specific advantages. First, it enables maximal exploration of the cystic duct, avoiding residual stones. Second, it eliminates prolonged T-tube placement, which can cause pain at the tube site, inconveniences in the activities of daily living and work, and even severe complications due to accidental dislodgement, Third, in most patients with secondary choledocholithiasis, the cystic duct lumen is dilated. Leveraging the expanded confluence and the advantages of ultrathin choledochoscopy, a microincision at the cystic duct confluence causes minimal damage to the common bile duct and facilitates convenient and highly successful stone exploration and removal.

Although this technique has some advantages, strict patient selection is essential. We believe the most appropriate indications are as follows: preoperative diagnosis of gallbladder calculus and secondary common bile duct stones, with a common bile duct diameter of 6 to 8 mm and no signs of acute cholangitis; no significant variation in cystic duct confluence; choledocholithiasis with ≤3 stones or sludge-like stones; and intact sphincter of Oddi function without edema or stricture.

In addition to selecting the appropriate patients, mastering the surgical techniques and operational details is crucial. The following key points are noteworthy. First, the gallbladder is dissected from the gallbladder bed using a combined antegrade and retrograde approach, using the gallbladder as traction to completely strip the connective tissue around the cystic duct and fully skeletonize the cystic duct up to the confluence, especially for tortuous and redundant cystic ducts because failure to do so may make incising the confluence difficult. Second, the cystic duct lumen is longitudinally incised using tissue scissors, gently dilated with a dissecting forceps, and then transversely cut open. Bipolar electrocautery can be used for hemostasis at sites of duct wall oozing. Third, Heister’s valve is the primary barrier preventing choledochoscope entry into the bile duct. It can be dilated or even incised with dissecting forceps to fully open Heister’s valve. Fourth, thin tissue scissors are used to sharply increase the confluence along the direction of the cystic duct confluence. The incision should be as small as possible while ensuring smooth choledochoscopic entry with a length that does not exceed one-third of the circumference. Fifth, the incision site should avoid blood supply vessels because excessive cutting may lead to significant bile duct injury and predisposition to biliary strictures. For oozing sites, gauze compression is preferred initially; if ineffective, 5-0 PDS-II sutures can be applied. Sixth, meticulously examine whether the Oddi’s sphincter opening is patent, checking for stenosis or edema. Repeated stimulation of Oddi’s sphincter should be avoided to prevent edema. Patency of the distal bile duct is essential for successful primary closure.^[16]^

A biliary fistula is one of the most common complications of primary bile duct suturing. It is not only related to patient nutritional status and cholangitis, but also to suturing techniques and suture material selection.^[17,18]^ In the experimental group, continuous suturing was performed using 5-0 PDS-II, starting from the lowest point of the incision and extending beyond it, with a margin of approximately 1.5 mm. Mucosa-to-mucosa hallux valgus suturing was performed and the upper knot was extended beyond the cystic duct ligation clip to ensure tight bile duct closure and prevent suture loosening. Finally, a gauze was used to check for bile staining at the incision site, with additional single stitches reinforced when necessary. Both the experimental and control groups in this study had 1 case each of biliary fistula, corresponding to incidence rates of 1.11% and 1.08%, respectively. All patients were quickly cured with unobstructed drainage, with no statistically significant difference between the 2 groups.

Postcholecystectomy syndrome severely affects the quality of life of patients after biliary tract surgery, with excessively long cystic duct remnants and residual cystic duct stones being non-negligible contributing factors.^[19]^ In the experimental group, complete skeletonization and full-thickness incision of the cystic duct were performed, effectively preventing this complication. Biliary strictures have long been the most feared complications among biliary surgeons. In recent years, most domestic investigators have considered a common bile duct diameter >8 mm or >10 mm as an essential criterion for primary closure.^[20,21]^ However, in our experimental group, the common bile duct diameter ranged between 6 and 8 mm, indicating a high risk for biliary stricture with direct anterior wall closure. By skillfully using the natural anatomical advantage of the dilated confluence area and through long-term follow-up, none of the patients have developed biliary stricture to date.

In summary, under strict case selection criteria, minimally invasive exploration and treatment of secondary common bile duct stones via microincision at the cystic duct confluence was safe and effective. This approach aligns with the principles of enhanced recovery after surgery (i.e., “ERAS”), and can prevent excessive residual cystic duct lengthening and retention of calculus in the cystic duct, thus demonstrating clinical utility.

In conclusion, under strict case selection, microincision and exploration via the cystic duct confluence for the treatment of secondary stones in the small-caliber common bile duct is safe and effective, conforms to the concept of enhanced recovery after surgery (i.e., “ERAS”), and can avoid excessive residual cystic duct length and cystic duct stone residue. However, this study still has several limitations. First, as a single-center retrospective study, case selection may be influenced by the patient source of our medical center, leading to a certain degree of selection bias. Second, although clear inclusion and exclusion criteria were established, there may still be unidentified confounding factors in the baseline data between the 2 groups, which might affect the accuracy of the results. Furthermore, the follow-up duration of this study is relatively short, which may limit our comprehensive evaluation of long-term complications and therapeutic efficacy. Therefore, subsequent multicenter, prospective studies with long-term follow-up are needed for further validation. It is believed that microincision and exploration via the cystic duct confluence for the treatment of secondary stones in the small-caliber common bile duct will be gradually promoted and applied.

## Author contributions

**Conceptualization:** Yifeng Huang.

**Data curation:** Jiansen Xie.

**Data analysis:** Gengsheng He.

**Writing – original draft:** Yifeng Huang.

**Writing – review & editing:** Gengsheng He.
